# Endoscopic Third Ventriculostomy in Previously Shunted Children

**DOI:** 10.1155/2013/584567

**Published:** 2013-07-28

**Authors:** Eva Brichtova, Martin Chlachula, Tomas Hrbac, Radim Lipina

**Affiliations:** ^1^Clinic of Pediatric Surgery, Orthopaedics and Traumatology, Brno Faculty Hospital, Cernopolni 9, 62500 Brno, Czech Republic; ^2^Clinic of Neurosurgery, Ostrava Faculty Hospital, 17. listopadu 1790, 70852 Ostrava-Poruba, Czech Republic

## Abstract

Endoscopic third ventriculostomy (ETV) is a routine and safe procedure for therapy of obstructive hydrocephalus. The aim of our study is to evaluate ETV success rate in therapy of obstructive hydrocephalus in pediatric patients formerly treated by ventriculoperitoneal (V-P) shunt implantation. From 2001 till 2011, ETV was performed in 42 patients with former V-P drainage implantation. In all patients, the obstruction in aqueduct or outflow parts of the fourth ventricle was proved by MRI. During the surgery, V-P shunt was clipped and ETV was performed. In case of favourable clinical state and MRI functional stoma, the V-P shunt has been removed 3 months after ETV. These patients with V-P shunt possible removing were evaluated as successful. In our group of 42 patients we were successful in 29 patients (69%). There were two serious complications (4.7%)—one patient died 2.5 years and one patient died 1 year after surgery in consequence of delayed ETV failure. ETV is the method of choice in obstructive hydrocephalus even in patients with former V-P shunt implantation. In case of acute or scheduled V-P shunt surgical revision, MRI is feasible, and if ventricular system obstruction is diagnosed, the hydrocephalus may be solved endoscopically.

## 1. Introduction

Endoscopic third ventriculostomy (ETV) is considered as a routine and safe method for obstructive hydrocephalus treatment. ETV is indicated in hydrocephalus with MRI proven obstruction in aqueduct or outflow parts of the fourth ventricle. For this indication, the ETV success rate reaches 90% [1, 2]. However, some patients with obstructive hydrocephalus were treated by ventriculoperitoneal (V-P) drainage (shunt) implantation even today. The most common reason is endoscopic treatment or acute MRI unavailability, or hyperacute hydrocephalus course. In case of V-P drainage failure in these patients, the possibility of subsequent endoscopic treatment and V-P drainage removal should be evaluated. The purpose of the study is to evaluate the success rate of ETV in the treatment of obstructive hydrocephalus in pediatric patients with previous V-P drainage implantation, with a view of subsequent V-P drainage removal.

## 2. Materials and Methods

**Figure 1:**
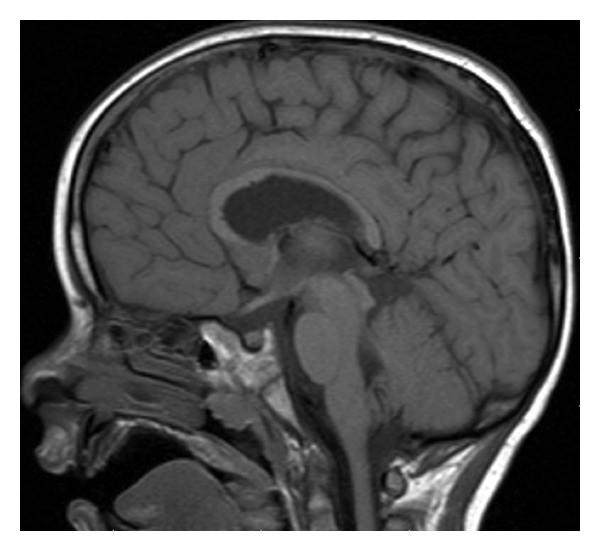
Preoperative MRI with apparent aqueductal obliteration.

**Figure 2:**
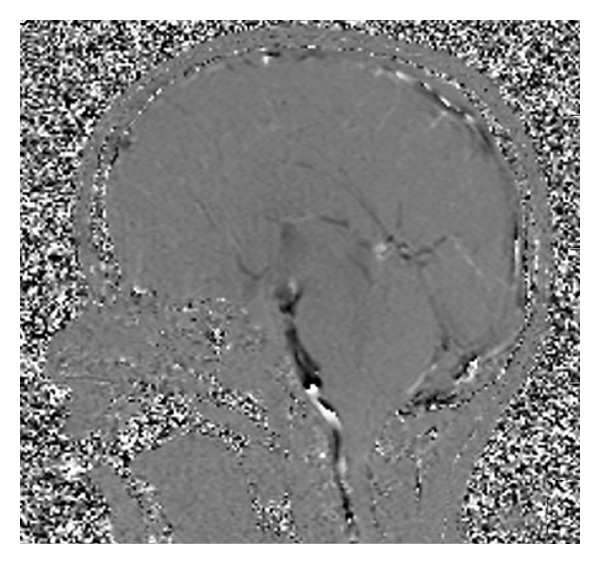
Postoperative MRI 2D phase contrast with third ventricle CSF flow evidence.

In the period of January 2001–December 2011, the ETV was performed in 42 patients with obstructive hydrocephalus, all with previous V-P drainage implantation. The group consisted of 24 boys and 18 girls; the mean age at the time of ETV was 9.5 years. The time between V-P drainage implementation and ETV ranged from 4 months to 12 years. There were 15 patients with congenital aqueduct stenosis, 15 patients with posthemorrhagic hydrocephalus, 7 patients with postinfectious hydrocephalus, and 5 patients with Chiari malformation associated hydrocephalus in our group. Unsuccessful ETV was performed in infancy in 9 patients (Figure 1). ETV was performed for acute hydrocephalus decompensation due to V-P drainage failure in 15 patients. In 5 patients, ETV was performed after V-P drainage removal and treatment due to drainage infectious complications (external ventricular drainage used till ETV performed), and in 22 patients, ETV was conducted instead of otherwise indicated extension of peritoneal drainage catheter. In 1 patient, ETV was performed as a planned surgery in full V-P drainage functionality. All patients were preoperatively examined by MRI with the evidence of aqueductal obstruction or obstruction in outflow parts of the fourth ventricle. In 13 patients, MRI was performed immediately before surgery, in the remaining 29 patients 1–15 months before the ETV. During the surgery, original V-P drainage was clipped first and then ETV was performed. In the case of normal V-P drainage ventricular catheter and reservoir functionality the drainage clipping was performed below the reservoir to allow cerebrospinal fluid aspiration. In the case of ventricular catheter dysfunction or reservoir absence, the Ommaya reservoir was inserted in 5 patients with acute hydrocephalus due to V-P drainage failure to enable emergency cerebrospinal fluid tapping. ETV itself was carried out using a rigid 6 mm diameter endoscope with direct optic (MINOP, B. Braun) or 3 mm diameter endoscope (PaediScope, B. Braun) in younger children and in case of narrow ventricle system. Neuroendoscopy Storz was used in 6 cases. Patients were hospitalized routinely for one week after the standard ETV. If in good clinical condition, patients were dismissed and then MRI was performed at intervals of 3 months (Figure 2). V-P drainage was subsequently removed if the following conditions were met: good clinical status, functional stoma, and lack of ventricular enlargement according to MRI performed 3 months after ETV. In 20 patients, the Ommaya reservoir or drainage ventricular catheter with original reservoir was left. Patients with removed V-P drainage and with no need of further V-P drainage introduction or other surgery for hydrocephalus 1 year after ETV were evaluated as successful. After V-P drainage removal, patients were followed up in pediatric neurologist and neurosurgeon outpatients. Postoperative follow-up algorithm includes pediatric neurologist and neurosurgeon examination every 6 months and since 2003 routinely two-year intervals MRI with a focus on late ETV failure radiological signs.

## 3. Results

Of the 42 pediatric patients, we were successful in 29 children in whom the V-P drainage was removed with no need of further V-P drainage introduction or any other surgery for hydrocephalus 1 year from ETV, and thus the overall success rate was 69%. We were most successful in patients with congenital aqueductal stenosis (12 of 15 patients, 80%); the worst results were obtained in the group of patients with postinfectious hydrocephalus (4 successful of 7 patients, 57%). In the group of 9 patients who had already underwent ETV in infant or neonatal period, the success rate 56% has been achieved (5 successful of 9 patients). Most of these patients suffered from posthemorrhagic obstructive hydrocephalus. Overall, we were unsuccessful in 13 children (31%). In 3 patients operated on for V-P drainage dysfunction, the drainage function had to be restored from 10 to 48 hours after ETV by replacing the dysfunctional part of drainage catether due to ETV dysfunction. In 6 children with planned ETV, we performed V-P drainage revision and functional restoration from 2 to 7 days after ETV. In 2 cases, we were forced to remove the drainage clip 8 and 20 hours, respectively, from ETV under local anesthesia. In 1 patient, the V-P drainage had to be reinserted after three weeks and in 1 patient after three months from the ETV. This patient had no clinical symptoms of intracranial hypertension, but missed MRI flow void phenomenon and ventricular system enlargement implicated V-P drainage restoration. There were 2 serious complications in our group of patients. Acute ETV failure developed in 1 patient after 1 year from ETV and in 1 patient after 2.5 years from V-P drainage removal. Clinical course was very similar in both ETV failure cases presenting a brief episode of weakness followed by sudden unconsciousness with bilateral mydriasis and respiratory failure. CT revealed acute hydrocephalus, and therefore acute external ventricular drainage was performed in both patients. However, the neurological findings did not improve after the procedure and comatose state persisted. Control CT scans revealed malignant cerebral edema, so bilateral decompressive craniectomy was performed in the second case. The neurological status deteriorated to the state of a reactive coma, and both patients died on the fourth day after acute ETV failure. The autopsy proved the signs of herniation through foramen magnum in both patients and histological examination of the 3rd ventricular floor proved closure of the stoma by gliotic tissue in the second case. One patient with minor complication required surgical revision for cerebrospinal fluid pseudocyst after ETV. No other complications occurred. Overall mortality rate was 4.7%. The results are summarized in Table 1.

## 4. Discussion

In the past, the V-P drainage or ventriculoatrial (VA) drainage insertion was the most commonly used method for hydrocephalus treatment, including obstructive hydrocephalus. The diagnostic possibilities provided by MRI, as well as the extension of endoscopic surgery in neurosurgery in general, introduced ETV in obstructive hydrocephalus surgery. Nowadays, ETV is the first choice for obstructive, noncommunicating hydrocephalus treatment with a success of rates up to 90% in case of aqueductal stenosis [2]. ETV advantage against V-P drainage is restoration of physiological cerebrospinal fluid (CSF) circulation, the absence of foreign material, and lower incidence of late complications [2, 3]. With the onset of hydrocephalus endoscopic treatment, it was questionable whether the endoscopy should be used in patients with obstructive hydrocephalus previously treated by V-A or V-P drainage. The original assumption that after a few years with working V-P drainage the lowering of CSF resorption capacity occurs has not been confirmed and the success of ETV in patients with previous V-P drainage implantation is comparable to the success rate of primary ETV [4]. In patients with the necessity of V-P drainage surgical revision, the ETV implementation carries the possibility of further V-P drainage removal, which was also the goal of our work. Our success rate was 69%, even if patients with postinfection and posthemorrhagic hydrocephalus in addition to MRI proven obstruction (with generally lower success rate due to possible CSF hyporesorption) were included. As reported in the literature, we have similar experiences with successful ETV in patients who underwent endoscopic surgery in infancy, and V-P drainage has been inserted due to initial ETV failure. Repeated ETV after a few years could be successful [5]. In our group, the success rate in these patients was 56%. The actual ETV technique in our group did not differ from the primary endoscopic surgery, and complications did not increase in comparison with the primary ETV. However, increased risk of intra- and postoperative ETV complications in patients with previous V-P drainage was referred to in the literature [6]. Woodworth et al. [7] point out to 2.5 times greater risk of ETV failure in patients with previous V-P drainage implantation. In our group, we had 2 serious complications and deaths due to late ETV failure [8]. Death in consequence of ETV failure, however, is not very common [8]. After analysing available data in published series of 13 patients [9–13], in 8 patients, a V-P shunt was implanted before the endoscopic procedure and ETV was performed due to failure of this shunt (based on radiologically proven obstructive hydrocephalus). In 4 patients without previously implanted V-P shunt, signs of acutely decompensated hydrocephalus were present. Stenosis of Sylvian aqueduct as the cause of hydrocephalus was determined in 8 patients and tectal gliomas in 2 patients. In 4 patients, the hydrocephalus was indicated as a congenital one. Sudden death occurred in 2 patients at home; in 9 patients, there was a rapid deterioration of consciousness with necessary intubation before or just after arrival to the hospital, all of them in a time course of several hours. The remaining 3 patients were admitted to the hospital for observation due to persisting headaches or vomiting; rapid deterioration developed here within several hours as well [8–13]. Both ETV failures in our group happened in first patients, where ETV was performed due to V-P drainage failure. Since then, we keep V-P drainage ventricular catheter with a reservoir or Ommaya reservoir in patients after ETV with previous acute V-P drainage dysfunction. This is also recommended by other authors in general at ETV [14]; some authors even recommend temporary external ventricular drainage in patients with previous V-P drainage [15]. We also perform regular MRI examinations every second year in all patients, even without clinical problems. Due to the fact that, even after the ETV, late failure can occur with the incidence of 2–15% [3, 16, 17], the question is whether previously shunted patients should be operated on as planned, or in case of V-P drainage failure. In our group, the majority of patients were operated on for either acute V-P drainage dysfunction or surgical revision necessity, mostly due to insufficient abdominal drainage catether length. Of course, the alternative solution was debated widely with parents. Only in one case, we performed ETV and V-P drainage clipping in totally asymptomatic patient with congenital aqueduct stenosis. The indication was supported by MRI proven obstruction and parental influence attempting to remove V-P drainage by means of ETV. However, the course was unsuccessful in this patient. We did not repeat similar indication and we cannot recommend it. Regarding the ETV success according to hydrocephalus etiology, the most successful we were in cases of congenital aqueductal stenosis, as expected. Conversely, the lowest success rate was recorded in patients with obstructive posthaemorrhagic hydrocephalus, with probably participating CSF hyporesorption. Is it possible to predict and to prevent a rapid deterioration leading to death after late failure of ETV? There are several factors contributing to the speed of formation of the symptoms: the type of hydrocephalus, time course of symptoms leading to ETV, and previous implantation of V-P shunt. Previous V-P shunt implantation with overdrainage manifestations may cause reduced ventricular wall compliance and increase of the elastance, thus elimination of compensation mechanisms during acute intracranial hypertension [8]. The rapidity of clinical course is affected by pathogenesis of the hydrocephalus as well. In case of obstructive hydrocephalus formed on Sylvian aqueduct stenosis basis, we can assume rapid exhaustion of brain compensation capacities with increased intracranial pressure. Another predicting factor is rapid progression of intracranial hypertension symptoms before the ETV was carried out [8].

## 5. Conclusions

Endoscopic third ventriculostomy is the method of choice for obstructive hydrocephalus treatment even in patients with previous V-P drainage implantation. Therefore, in case of acute or planned V-P drainage revision, MRI examination is advisable, and if obstruction in the ventricular system is diagnosed, it is possible to treat the hydrocephalus endoscopically. The possibility of endoscopic treatment should be considered already during the V-P drainage patients follow-up. Due to the possibility of late ETV failure, the outpatients follow-up is still necessary and also proper instruction of parents about this possibility.

## Figures and Tables

**Table 1:** Results according to obstructive hydrocephalus etiology.

Obstructive hydrocephalus etiology	Total number of patients	Previous ETV	Successful ETV	Complications
Congenital aqueductal stenosis	15	2	12 (80%)	1
Posthemorrhagic	15	6	10 (66%)	1
Postinfectious	7	1	4 (57%)	0
Chiari malformation	5	0	4 (80%)	0
